# Acute lung injury and acute respiratory distress syndrome

**DOI:** 10.4103/0974-2700.58663

**Published:** 2010

**Authors:** Maximillian Ragaller, Torsten Richter

**Affiliations:** Department of Anesthesiology and Intensive Care Medicine, University Hospital Carl Gustav Carus, Dresden, Germany

**Keywords:** Lung injury, ventilation-perfusion mismatching, protective ventilation, PEEP, recruitment

## Abstract

Every year, more information accumulates about the possibility of treating patients with acute lung injury or acute respiratory distress syndrome with specially designed mechanical ventilation strategies. Ventilator modes, positive end-expiratory pressure settings, and recruitment maneuvers play a major role in these strategies. However, what can we take from these experimental and clinical data to the clinical practice? In this article, we discuss substantial options of mechanical ventilation together with some adjunctive therapeutic measures, such as prone positioning and inhalation of nitric oxide.

## INTRODUCTION

Acute lung injury (ALI) and acute respiratory distress syndrome (ARDS) are still life-threatening diseases in critically ill patients. They are the manifestations of an inflammatory response of the lung to both direct and indirect insults and are characterized by severe hypoxemia, hypercapnia, diffuse infiltration in the chest X-ray, and a substantial reduction in pulmonary compliance. As part of the therapy for the underlying disease (such as shock, trauma, sepsis, pneumonia, aspiration, or burns), mechanical ventilation is critical for resolving life-threatening hypoxia and hypercapnia. However, studies from experimental and clinical fields have shown that mechanical ventilation, if performed incautiously, will further damage the lungs due to overinflation, barotrauma, and cyclic closing and reopening of the alveoli. This phenomenon has been named ventilator-associated lung injury (VALI). Furthermore, the mechanism of VALI can cause or trigger a pulmonary and systemic inflammatory reaction that may further lead to multiple organ dysfunction and multiple system organ failure.

Therefore, patients with ALI or ARDS need to be ventilated in a way that induces no further harm and avoids VALI. Hence, we offer an overview of evidence-based strategies of mechanical ventilation. Some adjunctive strategies that can modulate mechanical ventilation for the treatment of ventilator-associated pathologies will be discussed additionally.

## EPIDEMIOLOGY OF ALI AND ARDS

Using the American European Consensus definitions of ALI and ARDS [Table 1], epidemiological data regarding incidence rates show remarkable differences: 18–79 ALI cases/100,000 persons per year, versus 13–59 ARDS cases/100,000 persons per year. An actual epidemiological survey reports 190,000 cases per year in the United States. Despite much improvement in the understanding of its pathophysiology and recent success in therapy, the mortality rate remains high at 35–40%. ARDS mortality in trauma is relatively low (10–15%); the highest number of deaths was observed in patients with sepsis, pneumonia, or aspiration.[1,2]

**Table 1:** Definition of ALI/ARDS

Acute onset of the disease
Diffuse, bilateral infiltrate in chest X-rays consistent with pulmonary edema
No clinical evidence of left ventricular failure or elevated left arterial pressure, wedge pressure ≤ 18 mmHg, if measured
Hypoxemia: PaO_2_/FiO_2_ ≤ 300 mmHg = ALI
PaO_2_/FiO_2_ ≤ 200 mmHg = ARDS, regardless of the level of PEEP

PEEP: POSITIVE END-EXPIRATORY PRESSURE

## DEFINITION

ALI is defined as an acute lung disease with bilateral pulmonary infiltrate in a chest radiograph consistent with the presence of edema and no clinical evidence of left atrial hypertension; or (if measured) a pulmonary wedge pressure of 18 mmHg or less. Additionally, the ratio of arterial oxygen to the fraction of inspired oxygen (PaO_2_/FiO_2_) must be 300 mmHg or less, regardless of the level of positive end-expiratory pressure (PEEP). ARDS, the most severe form of ALI, is defined by a ratio of arterial oxygen to fraction of inspired oxygen of 200 mmHg or less [Table 1]. Although the term ARDS is often used interchangeably with ALI, by strict criteria, ARDS should be reserved for the most severe form of the disease.[3,4]

Although this definition is simple to apply in the clinical setting, it has been challenged over the years for several reasons. For example, it gives no information about the underlying disease and the interpretation of the nonspecific radiographic criteria may have high interobserver variability. Recent studies suggest that pulmonary ARDS seems to be quite different from nonpulmonary ARDS, and this may influence therapeutic strategy.[5]

A recent study categorized ALI and ARDS patients based on an early assessment of oxygenation on specific ventilator settings. Out of 170 patients with PEEP ≥ 10 cm H_2_O and FiO_2_ ≥ 0.5 for more than 24 h, 99 fulfilled ARDS criteria and experienced a mortality of 45.5%, whereas 55 patients had only ALI and experienced a mortality of 20%. This study demonstrates the large variability of the severity of the disease as well as the strong correlation between oxygenation impairment on day 1 and mortality.[6] Furthermore, Gattinoni and coworkers provided evidence of different responses to respiratory mechanics in ARDS of pulmonary versus extrapulmonary origin. Increasing PEEP to 15 cm H_2_O induced mainly overdistension in pulmonary ARDS, whereas in ARDS of extrapulmonary origin, PEEP was more effective in the recruitment of collapsed alveoli.[7] In pulmonary ARDS, consolidation of the alveoli by exaggerated inflammation is the predominant damage, whereas the formation of atelectasis is relatively rare. In extrapulmonary ARDS, alveolar collapse is more prominent; therefore, recruitment maneuvers (RMs) are probably more successful in these patients as demonstrated by Lim *et al*.[8] Especially in ARDS of extrapulmonary origin, it is advisable to observe lung and chest wall mechanics to classify the severity of ARDS and to optimize mechanical ventilation.[9]

## PATHOPHYSIOLOGY

A detailed description of the pathogenesis of ALI and ARDS on different levels (cellular, molecular, and so on) was published previously.[10–14] In brief, the alveolar-capillary unit is composed of the alveolar endothelium and the microvascular endothelium. Whatever insult is applied to the lung, it will result in more or less diffuse damage to this blood–gas barrier and, therefore, impair gas exchange. In pulmonary ARDS, the insult hits the alveolar endothelium primarily (e.g., pneumonia, aspiration), whereas in extrapulmonary ARDS (e.g., sepsis, pancreatitis, shock) the microvascular endothelium is the target. However, at a distinct point in the disease both entities react relatively uniformly with a diffuse inflammation of lung tissue. The host's redundant inflammatory network is the key factor in the development and progression of ARDS. Starting either from the alveolar or the microvascular side, the inflammatory process leads to alveolar and interstitial edema, reduced alveolar fluid clearance, impairment of surfactant production and function, and lung fibrosis. The persistent elevation of inflammatory mediators (mostly of neutrophil origin) in the broncho-alveolar-lavage precludes a resolution of the inflammatory process in the lungs. Due to gravity, in the supine position formation of edema is pronounced in dorsal basal areas, which leads to atelectasis, cyclic closing and reopening of alveoli, and loss of gas exchange lung surface. Moreover, this results in pronounced ventilation/perfusion mismatching, intrapulmonary shunting, pulmonary hypertension, reduced lung compliance, and global respiratory failure. The release of inflammatory mediators from damaged lung tissue triggers systemic inflammation (SIRS) and may lead to multiple organ failure, which is the main cause of death in ARDS patients.

At the bedside, ALI and ARDS culminate in life-threatening hypoxia, hypercapnia, acidosis and pulmonary hypertension, and require a fast and goal-oriented therapy without further lung damage.

## THERAPY STRATEGIES

Currently, there is no specific treatment for ARDS. Therefore, treatment strategies for ALI and ARDS must address the following three considerations:

1. Treatment of the underlying disease
2. Mechanical ventilation to secure oxygenation and CO_2_ elimination
3. Adjunctive procedures for the treatment of specific pulmonary pathologies

### Therapy of the underlying disease

ALI and ARDS describe a substantial impairment in gas exchange, but do not reveal the trigger of this syndrome. Therefore, further diagnostic procedures are often necessary to detect the underlying disease. An overview concerning the diseases frequently underlying ALI and ARDS and their specific therapy is given in Table 2. Without going into detail, it should be stated here that adequate treatment of trauma, shock, sepsis, or pneumonia is as critically important as mechanical ventilation. For example, early damage control surgery, early and effective antibiotics, rapid and sufficient hemodynamic stabilization guided by parameters of macro- and microcirculation are cornerstones of ARDS prevention as well as successful ARDS therapy [Table 2].

**Table 2:** Therapy for the underlying disease

Multiple trauma	Damage control surgery
Hemorrhagic shock	Fluid resuscitation, blood transfusion
Sepsis, infections general	Source control, antibiotics, fluid resuscitation
Pneumonia	Antibiotics
Aspiration	Bronchoscopy, antibiotics
Pancreatitis	Drainage, antibiotics
Near drowning	Surfactant
Burns, inhalation injury	Damage control surgery, fluid resuscitation, antibiotics, corticosteroids?
Cardiopulmonary bypass	Reduction of bypass time

### Mechanical ventilation

Mechanical positive-pressure ventilation with increased inspired oxygen concentration is lifesaving in patients with severe hypoxemia, diagnosed with ALI or ARDS. However, over the last two decades, mechanical ventilation has been oriented around false goals, for example, so-called normal values of the healthy.

The resulting high tidal volumes and high plateau pressures caused substantial harm to lungs and contributed to mortality. Ventilator-associated lung injury (VALI) can induce ALI and ARDS as well as multiple organ failure.[11,15–20] The different pathological mechanisms of VALI are shown in Table 3.

**Table 3:** Mechanisms of ventilator-associated lung injury

Barotrauma	Increased positive pressure (pmax/pplat)
Volutrauma	Over-distension of alveoli by high tidal volume (VT)
Mechanical shear stress	Cyclic closing and reopening of alveoli
Biotrauma	Inflammatory mediators released by shear stress
O_2_-toxicity	FiO_2_ > 0.6; toxic oxygen radicals

Lung damage caused by aggressive mechanical ventilation was demonstrated in many experimental studies and is supported by clinical observations.[21–25] This context led to a new concept of “protective ventilation.”

As interpreted today, this concept is more than a simple guideline for setting the ventilator. It is a comprehensive approach of dealing smoothly and carefully with a damaged lung that has an ongoing task to fulfill for the whole organism.

#### Goals of protective ventilation

With the protective ventilation concept, the goals of mechanical ventilation in the critical phase of ALI/ARDS are the focus, whereas one goal is the prevention of further harm to the lung. This means that mechanical ventilation has to provide sufficient oxygenation of the blood and should eliminate carbon dioxide to avoid respiratory acidosis and relieve the work of breathing. Once these vital aims are achieved, overdistension, barotrauma, atelectasis, hemodynamic impairment, and patient-ventilator asynchrony should be avoided. Therefore, it might not be necessary to achieve so-called normal values of oxygenation and CO_2_ clearance by using aggressive mechanical ventilation in an injured lung. As demonstrated by the ARDS-network study, a lower PaO_2_ during the first days was not associated with a higher morbidity and mortality rate.[22] From the early studies of Hickling and coworkers, we know that hypercapnia (i.e., permissive hypercapnia) has relatively low side effects as long as the kidneys can compensate the pH to acceptable values[26]. Therefore, a PaO_2_ of 60–80 mmHg (8–10.6 kPa), an oxygen saturation of ≥ 90%, and an increase in PaCO_2_ corresponding to a pH of >7.20 might be more appropriate.[22]

However, hypercapnia may cause elevated intracranial pressure, pulmonary hypertension, impairment of myocardial contractility, and decreased renal blood flow. Furthermore, patients with severe coronary artery disease might be unable to tolerate lower PaO_2_ values. The intensivist should keep these restrictions on the acceptability of abnormal blood gas values in mind while adjusting protective ventilation to the individual patient.

#### Protective ventilation: How to set the ventilator?

Conventional mechanical ventilation guided by normal values of gas exchange, with high tidal volumes (10–15 ml/kg body weight), a low-frequency to avoid death space ventilation, a tolerance of high inspiratory pressures, and a restricted use of PEEP was applied over several decades resulting in a mortality rate of 50–70%. This was challenged by a couple of small studies that used smaller tidal volumes and lower inspiratory plateau pressure to achieve a higher survival rate.[21,26] A milestone in the clinical development of protective mechanical ventilation was the ARDS network trial. In this PRC study, it was clearly demonstrated that a ventilation protocol using a tidal volume VT of 6 ml/kg predicted body weight (pbw) and a plateau pressure of max. 30 cm H_2_O and accepting lower PaO_2_ values resulted in a significantly lower mortality rate of 31% versus 39.8% (*P*=0.007) in the control group. Furthermore, a reduction in systemic inflammatory markers and an increase in ventilator-free days were demonstrated relative to a strategy using a VT of 12 ml/kg pbw.[22] A secondary analysis of the ARDS net database suggested that a further lowering of the plateau pressure (pplat) between 16 and 26 mbar was associated with a lower mortality.[27] One major criticism of this study was the setting of the PEEP using a fixed FiO_2_ coupling, which resulted in a mean PEEP of 9.6 cm H_2_O. However, this study demonstrated, for the first time in a large number of ARDS patients (n=861), a successful intervention relating to morbidity (higher number of ventilator-free days, lower number of organ failures) and mortality[22]. Regarding the correct choice of inspiratory oxygen concentration (FiO_2_), experimental data suggest that an FiO_2_ greater than 0.6 might be harmful to the lung tissue due to the production of free oxygen radicals and the suppression of anti-inflammatory pathways.[18,28,29] Based on these data, and in the absence of prospective studies in ARDS patients, most experts recommend an inspiratory oxygen concentration as low as possible to achieve the described goals of gas exchange.

These positive effects of protective ventilation in ALI and ARDS patients raise the question of whether this concept (VT 6 ml/pbw and pplat < 30 cm H_2_O) should be rolled out to all patients who are on mechanical ventilation, even if they do not meet ALI or ARDS criteria. Esteban and coworkers showed in a group of critically ill patients of miscellaneous origin (COPD, heart failure, pneumonia, trauma, etc.) that mortality was associated with the application of high VT and high pplat.[30]

Recently, Gajic *et al*. showed, in an observational study of more than 3000 critically ill patients with previously healthy lungs, that higher tidal volumes (>700 ml) and elevated pplat. (>30 mbar) were independent predictors of developing ALI and ARDS.[17] Putting these data together, it seems that even in the absence of prospective controlled randomized trials (PCRT) addressing this issue, it might be appropriate to apply the concept of protective ventilation to all critically ill patients who are on mechanical ventilation. Interestingly, an early study on the relation between lung volume and body weight in mammals showed a natural tidal volume of approximately 6.3 ml/kg.[31]

Protective mechanical ventilation is beneficial not only for the lungs but also for the heart. Positive-pressure ventilation with high tidal volumes and high plateau pressures including a PEEP is associated with increased transpulmonary pressure that results in a substantial reduction of right ventricular preload.[32] On the other hand, elevated transpulmonary pressure increases right ventricular afterload, which might already be elevated by hypoxic pulmonary vasoconstriction (due to atelectasis) and hypercapnia.[33,34] Both mechanisms lead to substantial reductions in cardiac output and blood pressure. In the worst case, this constellation will end in acute right ventricular failure (acute cor pulmonale). Therefore, using a low tidal volume and limited plateau pressure may unload the right ventricle resulting in a lower rate of cor pulmonale.[35] Furthermore, use of PEEP as discussed further may increase the gas exchange area (by a continuous but gentle recruitment of alveoli) and reduce hypoxic pulmonary vasoconstriction with a successive reduction in right ventricular afterload.

In conclusion, protective mechanical ventilation with a tidal volume of 6 ml/kg pbw, plateau pressure kept below 30 cm H_2_O, FiO_2_ as low as possible, and a permissive hypercapnia to a pH level of 7.2 is the key to successful ARDS therapy.[22,24,27] In patients without ALI or ARDS, protective ventilation is recommended as well to avoid VALI and ARDS.

For the calculation of predicted body weight, use the following formulas:

Man:    50 +0.91 × (body height in cm − 152.4)

Woman:  45.5 +0.91 × (body height in cm − 152.4).

#### Titration of PEEP

In patients with severe lung injury, the inflammatory response at the alveolar-capillary unit determines interstitial and intra-alveolar edema and atelectasis. Additionally, the increased lung weight due to fluid accumulation causes alveolar collapse, especially in the dependent areas, for example, in the dorsal basal areas of the lungs (in supine position). At the end of expiration, when intrapulmonary pressure is low, further derecruitment of alveoli will occur, which reduces the area of gas exchange and increases transpulmonary shunting. In the borderline zone, between already collapsed alveoli and normal ventilated alveoli, a cyclic recruitment and derecruitment of alveoli enhances the mechanical stress on lung tissue and maintains the inflammatory process.[36–38] These harmful mechanisms should be antagonized, by using positive intrapulmonary pressure at the end of expiration (PEEP). The implementation of PEEP leads to improvements in oxygenation, compliance, and functional residual capacity. Probably the most protective effect of PEEP is the prevention or minimizing of cyclic recruitment and derecruitment, which lessens mechanical distress and inflammation. Hence, PEEP has become a cornerstone in protective mechanical ventilation.[10,37–42]

However, despite its fundamental prominence, the issue of the best or optimal PEEP is still a matter of debate. Beyond that, side effects such as decreased cardiac output, reduced pulmonary perfusion, and pulmonary lymph drainage might be considered when titrating PEEP.[24,40]

Patients with ARDS were traditionally ventilated with PEEP levels between 5 and 12 cm H_2_O, and these were set according to the preferences of the individual intensivist.[30] In the ARDS network study mentioned earlier, the PEEP setting was determined by a fixed relationship between FiO_2_ and PEEP, as shown in Table 4. This relatively simple method of PEEP titration led to mean PEEP level of about 9.4 cm H_2_O. However, this fixed combination has been criticized owing to its lack of provision for the individual situation in a given patient.

**Table 4:** FiO_2_–PEEP relationship by ARDS network[22]

FiO_2_	0,3	0,4	0,5	0,6	0,7	0,8	0,9	1,0
PEEP	5	5–8	8–10	10	10–14	14	14–18	18–24

In certain experimental and small clinical trials, PEEP levels were titrated above the lower inflection point of the quasistatic pressure-volume curve (PV curve) of the respiratory system.[21,43] The lower inflection point of the inspiratory PV curve represents the pressure level at which all potentially recruitable alveoli are fully reopened. Setting the PEEP at 1 or 2 cm H_2_O above this level should keep the alveoli open at the end of expiration. Using this strategy, two studies with a significantly higher PEEP than in the ARDSnet study showed a favorable outcome.[21,43] Unfortunately, a lower inflection point cannot be detected easily in the PV curve of all ARDS patients in clinical settings; therefore, this technique remains experimental at the moment.[44]

Another more practical method for optimizing PEEP might be individual PEEP titration guided by PaO_2_, PaCO_2_, and compliance of the respiratory system. Accordingly, PEEP should be increased in steps of 2 cm H_2_O until the best oxygenation and compliance is achieved. Unfortunately, PaO_2_ does not always correlate with recruitment, which is why several experts propose the lowering of PaCO_2_ as a more informative value regarding recruitment of alveoli.[36] Best PEEP is, therefore, defined as best oxygenation and compliance at lowest PaCO_2_. An increase in PaCO_2_ represents an increase in death space ventilation of nonperfused but overdistended lung regions.[45,46]

Additionally, PEEP effects might be different in pulmonary and nonpulmonary lung injury. As pointed out by Gattinoni and coworkers, patients with pulmonary ARDS have stiffer lungs and more consolidated lung areas that are less responsive to PEEP, whereas in nonpulmonary ARDS atelectatic lung regions are usually more responsive to higher PEEP.[7]

Using these methods for setting the PEEP, one has to keep in mind that PEEP improves only gas exchange and alveolar ventilation, and it may have decremental effects on tissue oxygenation by reducing cardiac output and oxygen delivery. Therefore, one has to consider hemodynamic stability while titrating PEEP.[46–48] Up to now, there have been three prospective randomized controlled trials comparing higher PEEP levels with the PEEP levels used in the original ARDS network trial (mean 9.4 cm H_2_O). The study of Villar *et al*. showed beneficial effects of higher PEEP on morbidity and mortality.[43] Two other trials with 549 and 767 enrolled patients reported no improvement in survival due to mean PEEP levels of 13±3.5 or 15.8±2.9 cm H_2_O, respectively, in ALI or ARDS.[40,49] An actual meta-analysis including data from 2484 patients showed a trend toward reduced mortality with higher PEEP even though the difference did not reach statistical significance (odds ratio 0.90 CI 0.72–1.02; *P* = 0.077). The reduction in absolute risk of death was approximately 4%.[42]

In conclusion, for the routine patient with ALI, setting PEEP according to the ARDS net table is probably sufficient. In severe ARDS cases, individual titration of higher PEEP by blood–gas analysis, compliance measurement, and hemodynamic monitoring, or by identification of the LIP on the PV curve should be more appropriate.

#### Ventilatory modes

In addition to using the fundamental strategy of protective ventilation, the question of the optimal ventilatory mode might also be an important issue. As listed in Table 5, there are many different ventilators and many distinct ventilation modes available on the market. Basically, one has to distinguish between a volume- or pressure-controlled mode. Furthermore, there are many modes of controlled ventilation combined with distinct modes of augmented spontaneous breathing. However, there is little evidence regarding their specific influence on morbidity or mortality in patients with respiratory failure. In the large randomized trials on mechanical ventilation in ALI/ARDS patients, a volume-controlled or volume-assisted mode was usually applied.[21,22,40,43,49] Comparing a volume-controlled with a pressure-controlled mode, Esteban and coworkers did not find differences regarding mortality or morbidity.[50] Therefore, the mode of mechanical ventilation is less important than the strategy of protective ventilation regarding tidal volume, peak pressure, and PEEP. Moreover, a careful adjustment of alarm limits such as peak airway pressure and lower threshold of minute ventilation volume should be applied.

**Table 5:** Modes of mechanical ventilation

Controlled	Controlled	Spontaneous
volume-controlled V-CMV	pressure-controlled P-CMV	pressure support ventilation PSV/ASB proportional assist ventilation PAV
Inversed ratio ventilation	Inversed ratio ventilation	Synchronized intermittent mandatory ventilation
	Acute pressure release ventilation	Biphasic airway pressure ventilation BiPAP®, BiVent®, BiLevel®
	Biphasic airway pressure ventilation	Continuous positive airway pressure
	BiPAP®, BiVent®, BiLevel®	

#### Recruitment of alveoli

As described previously, ALI and ARDS are characterized by a more or less pronounced loss of gas exchange area due to collapsed and consolidated alveoli. Since the famous quote of Lachmann “open the lung and keep the lung open,” RMs are used to reopen these alveoli for gas exchange.[51] Up to now, there have been a significant number of different methods discussed in the literature [Table 6]. Most of them are based on a short but substantial increase in the inspiratory pressure that should improve ventilation in poorly aerated alveoli or reopen collapsed alveoli during inspiration. By applying a PEEP higher than the derecruitment threshold, these lung areas should be kept open. An RM usually improves oxygenation (PaO_2_/FiO_2_) and might have an influence on ventilation by reducing PaCO_2_.[45,47,51–55] As demonstrated in several animal models, recruitment occurs during the whole period of inspiration, and the amount of recruited lung area correlates with the inspiratory pressure applied.[21,51,56,57] However, CT scans of ALI and ARDS lungs showed high variability in alveolar recruitment in response to a standard RM. Interestingly 24% of the lungs, particularly the dependent areas of the lungs, could not be recruited by an RM applied with an inspiratory pressure of 45 cm H_2_O. Furthermore, the amount of recruited lung tissue correlated well with the severity of ARDS.[45] Moreover, earlier data suggest that an RM performed in the early phase of ARDS might be more effective than in late-stage ARDS.[47,58]

**Table 6:** Recruitment maneuvers

Pmax = 40 cmH_2_O for 40 sec Pmax up to 80 cmH_2_O; up to 120 sec PEEP > LIP or LIP+2 Intermittent, repetitive sigh VT 800–1000 ml High frequency oscillation ventilation Inversed ratio ventilation Prone positioning Spontaneous breathing

Due to the individual situation of a patient, an RM might cause deterioration in gas exchange, hyperinflation, and/or barotrauma.[46,54,59,60] On the hemodynamic side, the acute and substantial increase in intrathoracic pressure often induces a critical decline in cardiac output and tissue oxygenation and might result in right ventricular failure. An elegant study in the saline washout model proposed a so-called slow low pressure RM (PEEP=15 cm H_2_O + inspiratory hold for 7 sec twice every 15 min). This method was associated with a greater improvement in oxygenation, shunting, and compliance, whereas less circulatory depression was observed when compared to high-pressure maneuvers.[48] Therefore one has to stabilize the patient hemodynamically prior to an RM procedure.[47,61,62] Recently, a prospective multicenter trial with 967 patients enrolled showed no advantage with respect to mortality of a protective ventilation strategy including an RM if compared to the protective strategy alone. However, the number of hypoxic crises and the need for other rescue procedures like nitric oxide (NO) or extracorporeal membrane oxygenation (ECMO) were reduced in the recruitment group.[63]

In conclusion, neither has a standard RM been established nor is the application of an RM generally accepted in the critical care community. If a RM needs to be performed to resolve hypoxia, one has to first secure hemodynamic stability. The procedure itself should be performed gently with the lowest effective pressure.

#### Spontaneous breathing

It has been known since the early seventies that mechanical ventilation in the supine position and sedation lead to an upward shift of the diaphragm, especially in the dorsal basal regions due to pressure from the abdominal compartment (liver, spleen). This leads to a reduction in functional residual capacity (FRC) and might cause atelectasis in these areas, even in healthy lungs.[64] As a result, positive-pressure ventilation is more pronounced in the ventral lung areas. A sufficient contraction of the diaphragm, as provided by spontaneous breathing, may physiologically reopen atelectatic lung regions in the dorsal areas. Furthermore, spontaneous breathing reduces intrathoracic pressure, improves ventilation-perfusion-relations, and improves gas exchange without a critical increase in oxygen consumption.[65–67] As side effects of spontaneous breathing, sedation will be reduced and tissue atrophy of the respiratory muscles, which occurs after as little as 18 h of mechanical ventilation, might be diminished.[68]

Putting this all together, a trial of spontaneous breathing seems to be a physiological RM that is justified even in the early phase of ALI or ARDS.

Based on the data and conclusions discussed earlier, Table 7 gives a kind of master plan for mechanical ventilation during respiratory failure and in critically ill patients in general.

**Table 7:** Master plan of mechanical ventilation

Goals of mechanical ventilation	Concept of protective ventilation
PaO_2_: 8–10.6 kPa/60–80 mmHg	Avoid O_2_-toxicity, volu-, baro-, biotrauma
SaO_2_ ≥ 90%	Plateau pressure≤ 30 cm H_2_O
Permissive hypercapnia, PaCO_2_ as long as pH > 7.2	Tidal volume of 6 ml/kg predicted body weight Keep the alveoli open by PEEP Try spontaneous breathing early

### Adjunctive therapeutic options

In patients with ALI and ARDS, there are sometimes clinical situations where the intensivist has to augment his standards to surmount hypoxic crisis and to treat specific pathologies in the course of the disease. These adjunctive therapeutic procedures are indicated in specific cases only and should be recommended on an individual basis.

To resolve a hypoxic crisis, there are four measures available: RMs as pointed out previously, prone positioning, inhalation of NO, and ECMO.

#### Prone positioning

Due to the human anatomy, more lung tissue is located in the dorsal areas than in the ventral areas. Pictures from magnet resonance imaging (MRI) or CT scans show a more pronounced compression of central lung regions in the supine than in the prone position.[69,70] Upon putting a patient into the prone position, dorsal basal lung areas were relieved of the weight of the injured lung itself, and on the left side, from the weight of the heart as well. Usually this results in alveolar recruitment and an improvement in oxygenation and ventilation without the need for higher inspiratory pressures or increased mechanical stress on the lung tissue.[71,72] Additionally, regional ventilation–perfusion matching will be improved and transpulmonary shunting will be lessened.[73] In several clinical trials, prone positioning led to an improvement in oxygenation, ventilation, and compliance, and to a reduction in the inspiratory pressure or PEEP.[74–77] Unfortunately, this improvement in parameters of pulmonary function did not reduce mortality in ARDS or ALI patients. Only a subgroup of very severe ARDS patients (PaO_2_/FiO_2_ < 100 mmHg) and a patient-group in which a PaCO_2_-reduction took place after prone positioning benefited from this procedure.[75,77]

### Inhalation of nitric oxide

NO, identified as endothelium-derived relaxing factor (ERDF) in 1987, is the physiological vasodilator in the body. Inhaled NO, which is distributed only to the ventilated lung regions, leads to a relaxation of pulmonary vascular smooth muscle, thereby decreasing pulmonary hypertension and increasing the right ventricular ejection fraction. Selective vasodilation of well-ventilated lung regions should cause a shift of pulmonary blood flow to these alveoli, improving the matching of ventilation and perfusion and improving oxygenation during ARDS in about 80% of patients. Due to the rapid binding of the molecule to hemoglobin, systemic vasodilatation and hypotension should be avoided.[78–80] However, these effects are unfortunately transient and without any influence on mortality as demonstrated in recent trials.[81–83]

As shown in Table 8, there are further ALI and ARDS pathologies that might lead to experimental therapeutic interventions during mechanical ventilation.

**Table 8:** Adjunctive therapeutic options

Pathology	Adjunctive therapeutic options
Hypoxic crisis	Prone positioning
	Recruitment maneuver
	Inhaled NO,
	Extra corporal membrane oxygenation
Pulmonary hypertension	Inhaled NO, inhaled PGI_2_
IRDS, near drowning	Surfactant
Fibrosis	Corticosteroids

ECMO, PGI_2_, corticosteroids

Further adjunctive therapeutic procedures, such as aerosolized prostacyclin (PGI_2_) to reduce elevated pulmonary arterial pressure, corticosteroids to minimize the development of fibrosis of lung tissue in late ARDS, or inhaled perfluorocarbons are not evidence based at the moment. Administration of surfactant endotracheally in cases of surfactant deficiency, in such situations as near-drowning and infant respiratory distress syndrome (IRDS), may be helpful. The highly specialized equipment and knowledge required to provide ECMO made this technique available only in special medical centers, and it is therefore an alternative procedure. However, it could not decrease the mortality rate of severe ARDS.[84–86]

## CONCLUSION

The simple procedure of lung-protective ventilation, using reduced tidal volumes (6 ml/kg pbw), a pressure limit (below 30 cm H_2_O), and a FiO_2_ as low as possible is (currently) the only therapy whereby the mortality rate of patients with ALI/ARDS can be effectively reduced. Therefore, the consistent use of lung-protective ventilation has priority over all other therapeutic options. In doing so, VALI has to be definitively avoided.
